# Supplementary material for: Identification of regulators of poly-ADP-ribose polymerase inhibitor response through complementary CRISPR knockout and activation screens
Source: Nat Commun. 2020 Nov 30;11:6118. doi: 10.1038/s41467-020-19961-w (PMC7704667; doi:10.1038/s41467-020-19961-w)
Supplement: Supplementary file 1 — Supplementary Information [file 41467_2020_19961_MOESM1_ESM.pdf]

Supplementary Information

Identification of regulators of poly-ADP-ribose polymerase inhibitor response through complementary CRISPR knockout and activation screens

Clements et al.

Supplementary Figure 1

a

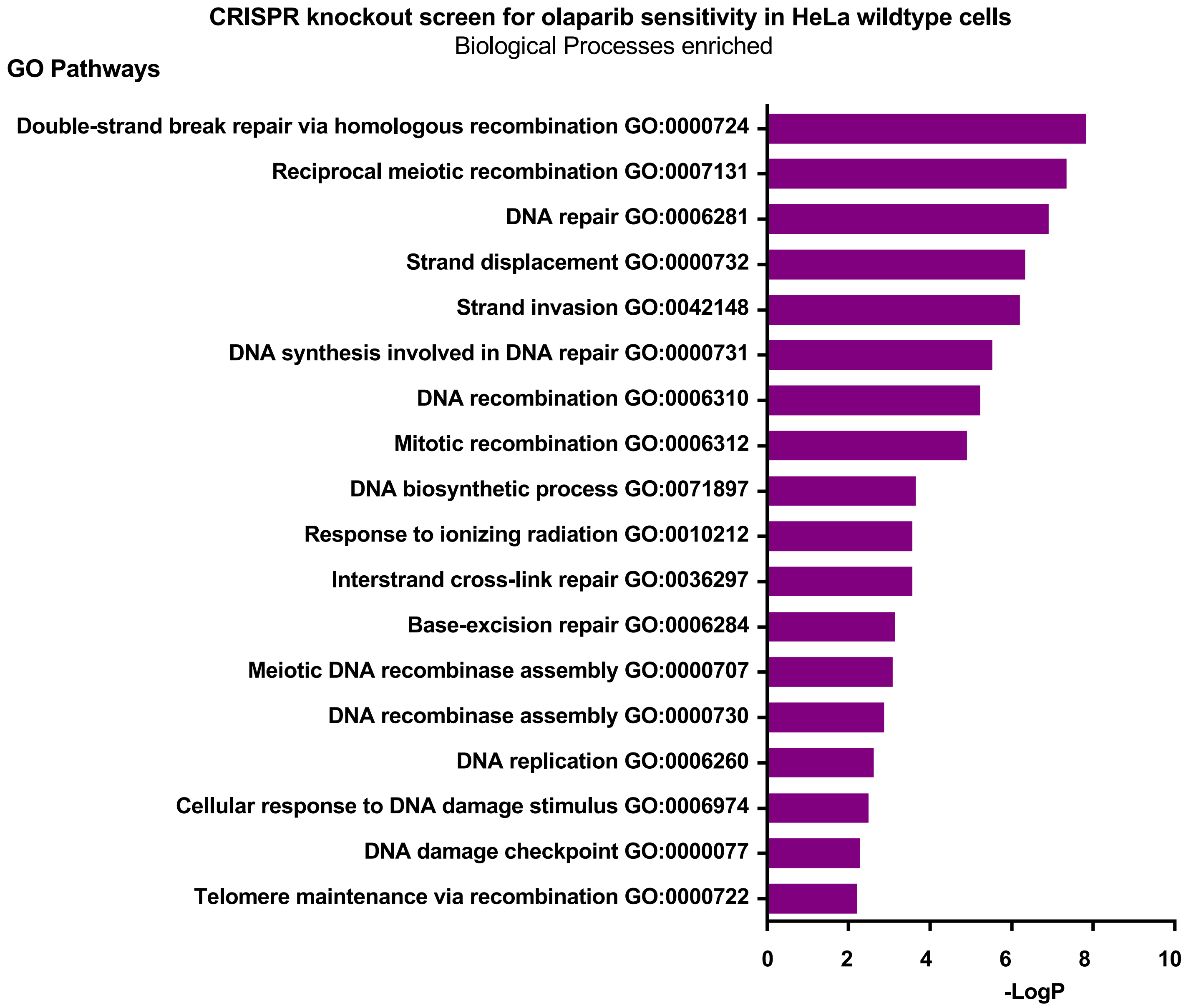

b

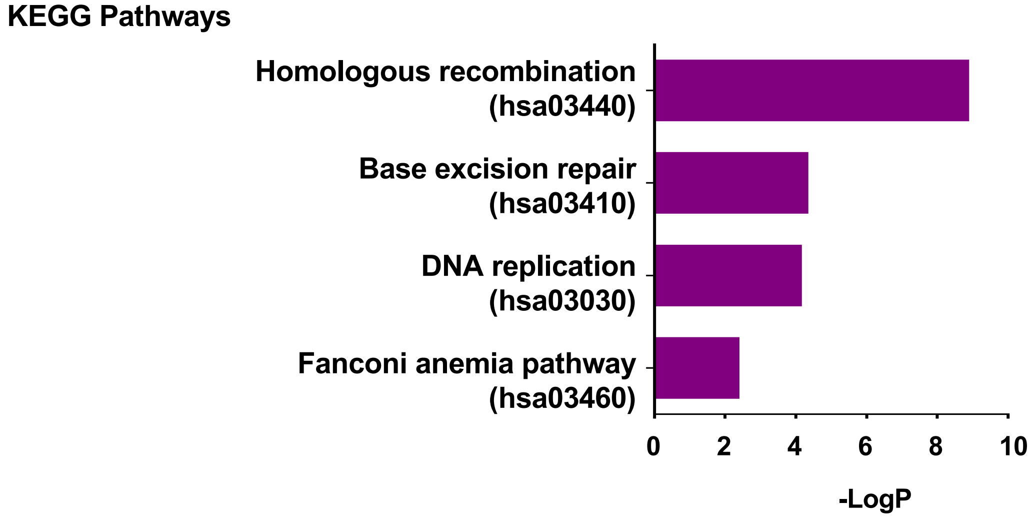

Supplementary Fig. 1. Gene ontology analysis of the top candidates from the CRISPR knockout screen for olaparib sensitivity in wildtype cells. Top hits with Log(score negative selection) less than -2.5 as calculated by MAGeCK were entered into NIH DAVID and pathways were analyzed for Gene Ontology Biological Processes (GO_BP) terms (**a**) or KEGG_PATHWAY terms (**b**). GO_BP and KEGG_PATHWAY terms with a LogP of -2 or lower are shown. Source data are provided as a Source Data file.

Supplementary Figure 2

a

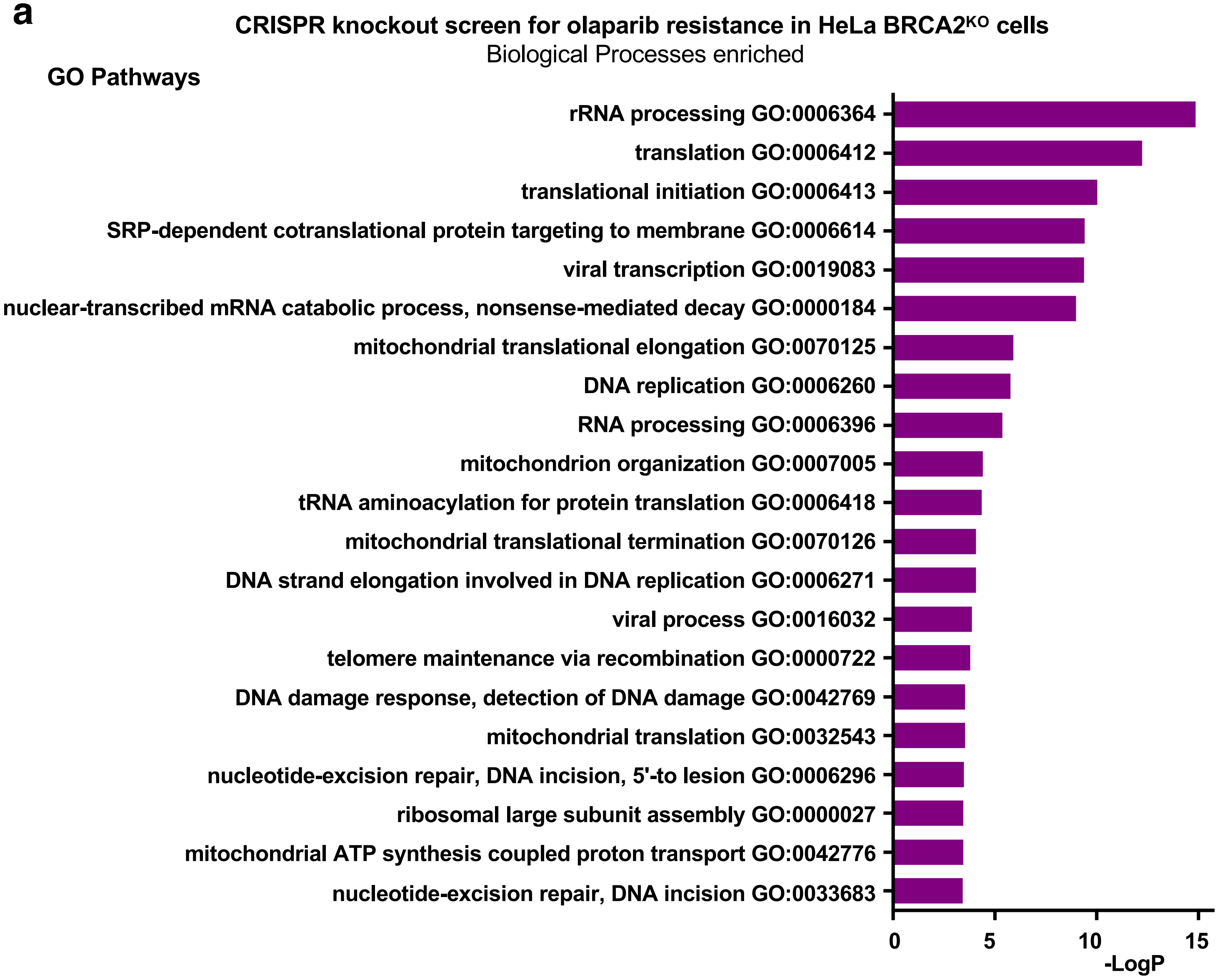

b

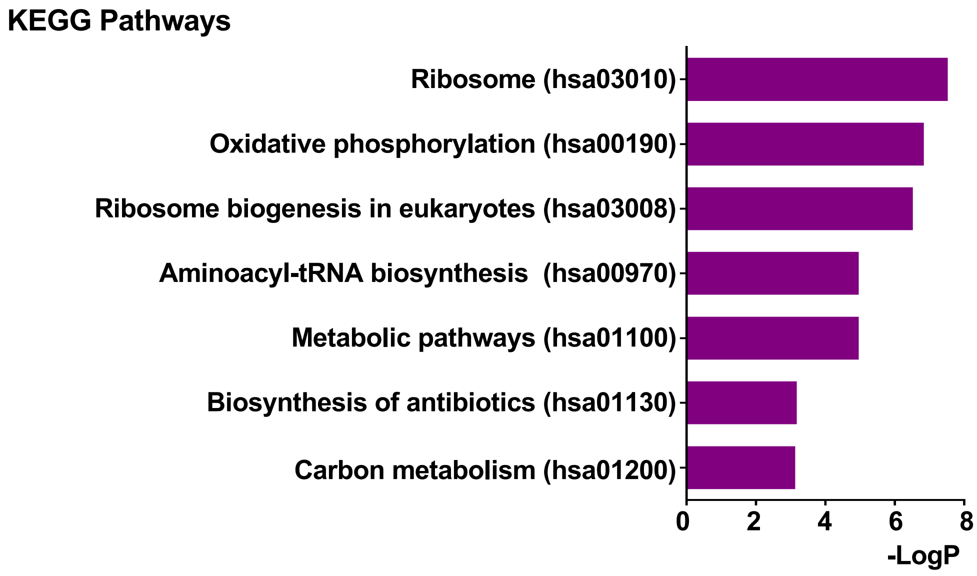

Supplementary Fig. 2. Gene ontology analysis of the top candidates from the CRISPR knockout screen for olaparib resistance in $BRCA2^{KO}$ cells. Top hits with Log(score positive selection) less than -3.5 as calculated by MAGeCK were entered into NIH DAVID and pathways were analyzed for GO_BP terms (**a**) or KEGG_PATHWAY terms (**b**). GO_BP and KEGG_PATHWAY terms with a LogP of -3 or lower are shown. Source data are provided as a Source Data file.

Supplementary Figure 3

a

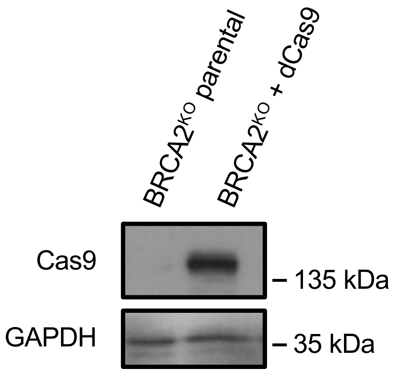

b

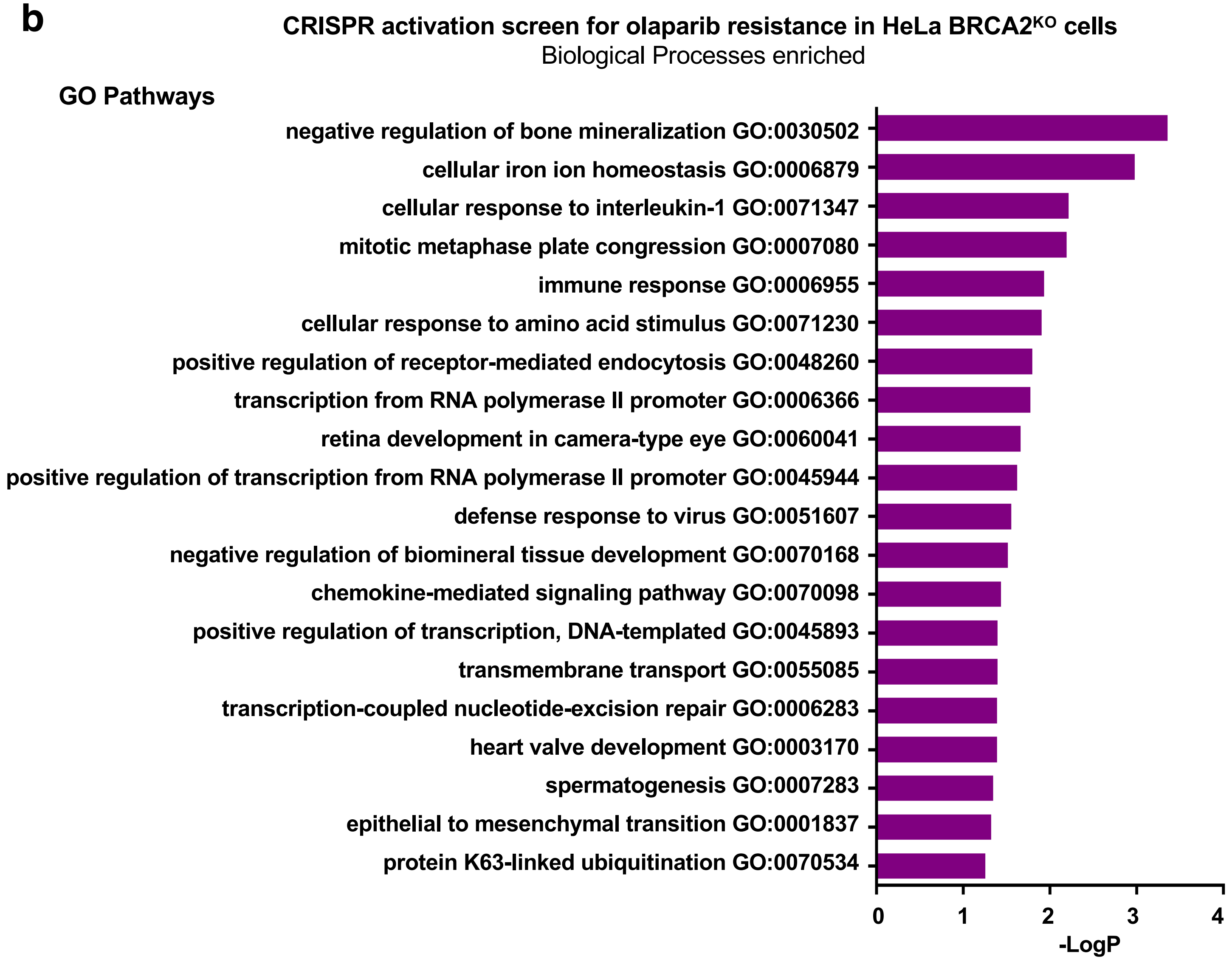

c

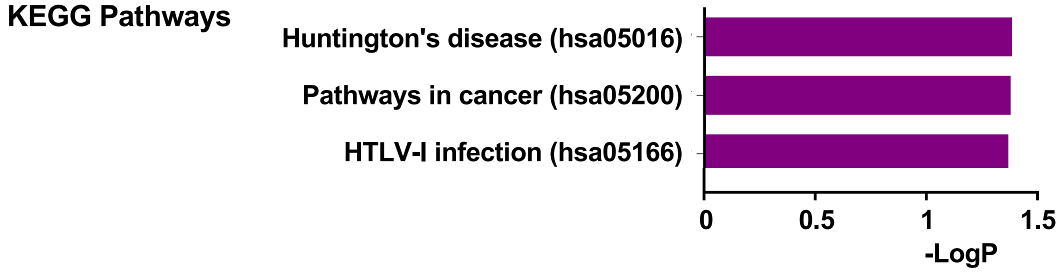

Supplementary Fig. 3. Gene ontology analysis of the top candidates from the CRISPR activation screen for olaparib resistance in BRCA2^{KO} cells. a. Western blot confirming expression of dCas9 in HeLa BRCA2^{KO} cells. **b, c.** Hits with Log(score positive selection) less than -2.5 as calculated by MAGeCK were entered into NIH DAVID and pathways were analyzed for GO_BP terms (**b**) or KEGG_PATHWAY terms (**c**). GO_BP and KEGG_PATHWAY terms with a LogP of -1.25 or lower are shown. Source data are provided as a Source Data file.

Supplementary Figure 4

a

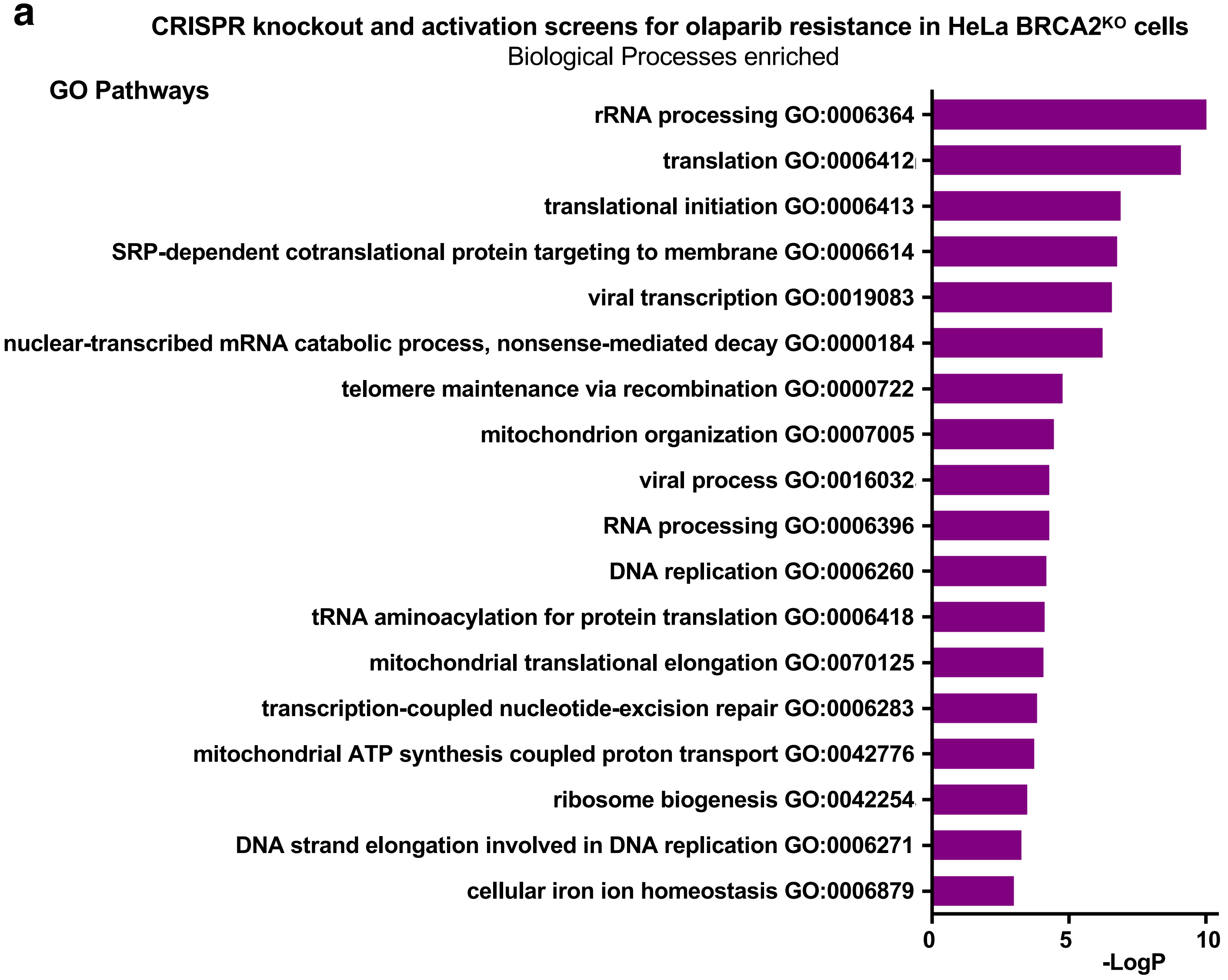

b

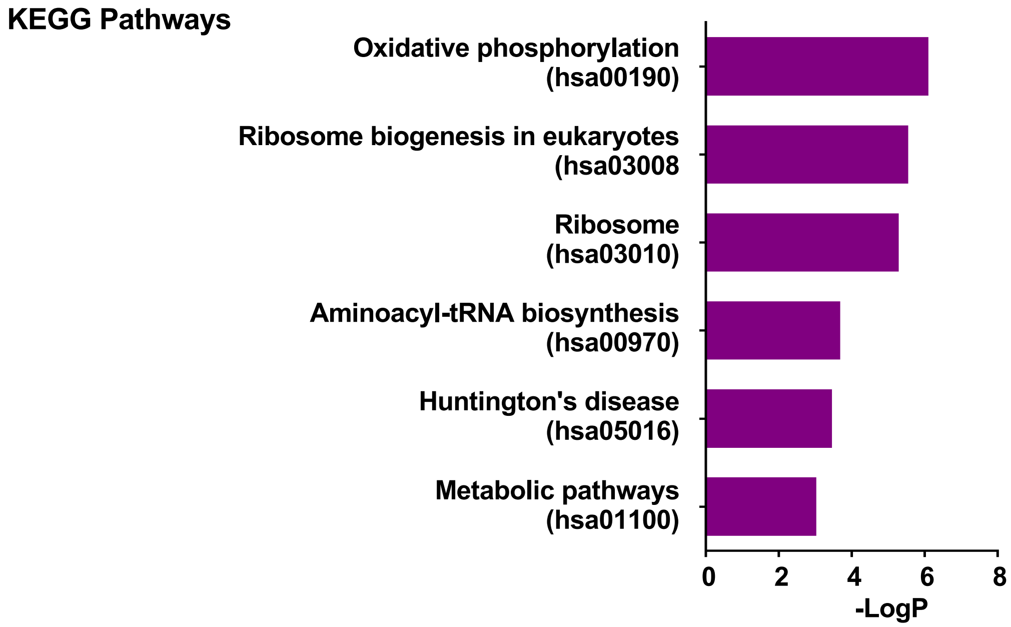

Supplementary Fig. 4. Gene ontology analysis of the top candidates pooled from the CRISPR knockout and activation screens for olaparib resistance in BRCA2^{KO} cells. Top hits with Log(score positive selection) lower than -3.5 from the knockout screen and lower than -2.5 from the activation screen, as calculated by MAGeCK, were pooled and entered into NIH DAVID and pathways were analyzed for GO_BP terms (**a**) or KEGG_PATHWAY terms (**b**). GO_BP terms and KEGG_PATHWAY terms with a LogP of -3 or lower are shown. Source data are provided as a Source Data file.

Supplementary Figure 5

CRISPR knockout screen for olaparib resistance in HeLa BRCA2^{KO} cells

DNA replication and repair genes among the most highly significantly enriched hits

**C10ORF2**	**HUWE1**	**PRIM1**
**CARM1**	**KAT5**	**PRMT1**
**DUT**	**MRPS35**	**RAD9A**
**E2F7**	**MTOR**	**RPA3**
**FEN1**	**NUP98**	**SUPT16H**
**GINS1**	**PARP1**	**UBA52**
**GINS2**	**POLD2**	**XRCC3**
**GTF2H1**	**POLG2**	

Supplementary Fig. 5. DNA replication and repair genes which were most highly significantly enriched in the PARPi resistance knockout screen in BRCA2^{KO} cell. The 23 genes associated with DNA repair or replication GO_BP processes among the top 214 hits with FDR value lower than 5% are listed.

Supplementary Figure 6

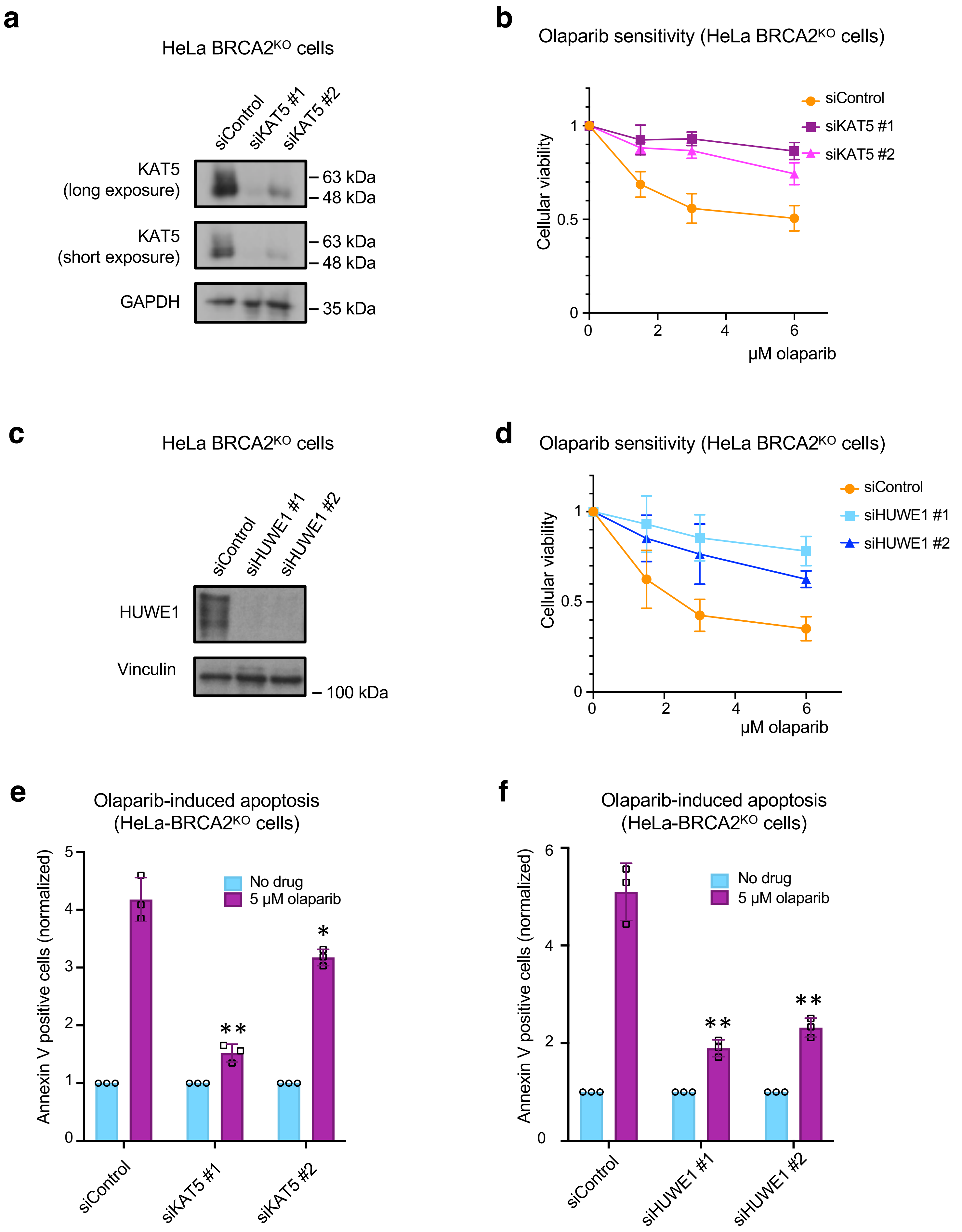

Supplementary Fig. 6. Knockdown of KAT5 or HUWE1 with either of two siRNA oligonucleotides causes PARPi resistance in HeLa BRCA2-knockout cells. **a.** Western blot showing that KAT5 is efficiently depleted with siRNA oligonucleotides employed. **b.** Depletion of KAT5 by two siRNA oligonucleotides rescues PARPi sensitivity in HeLa BRCA2-knockout cells in cellular viability assays. **c.** HUWE1 protein is depleted using two siRNA oligonucleotides as shown by western blot. **d.** Depletion of HUWE1 by two siRNA oligonucleotides rescues PARPi sensitivity in HeLa BRCA2-knockout cells in cellular viability assays. **e, f.** Olaparib-induced apoptosis is rescued by KAT5 **(e)** or HUWE1 **(f)** depletion in BRCA2-knockout cells. Asterisks indicate statistical significance (t-test, two-tailed, unequal variance) compared to the olaparib-treated siControl condition. All data are shown as averages of three experiments with standard deviations as error bars. Source data are provided as a Source Data file.

Supplementary Figure 7

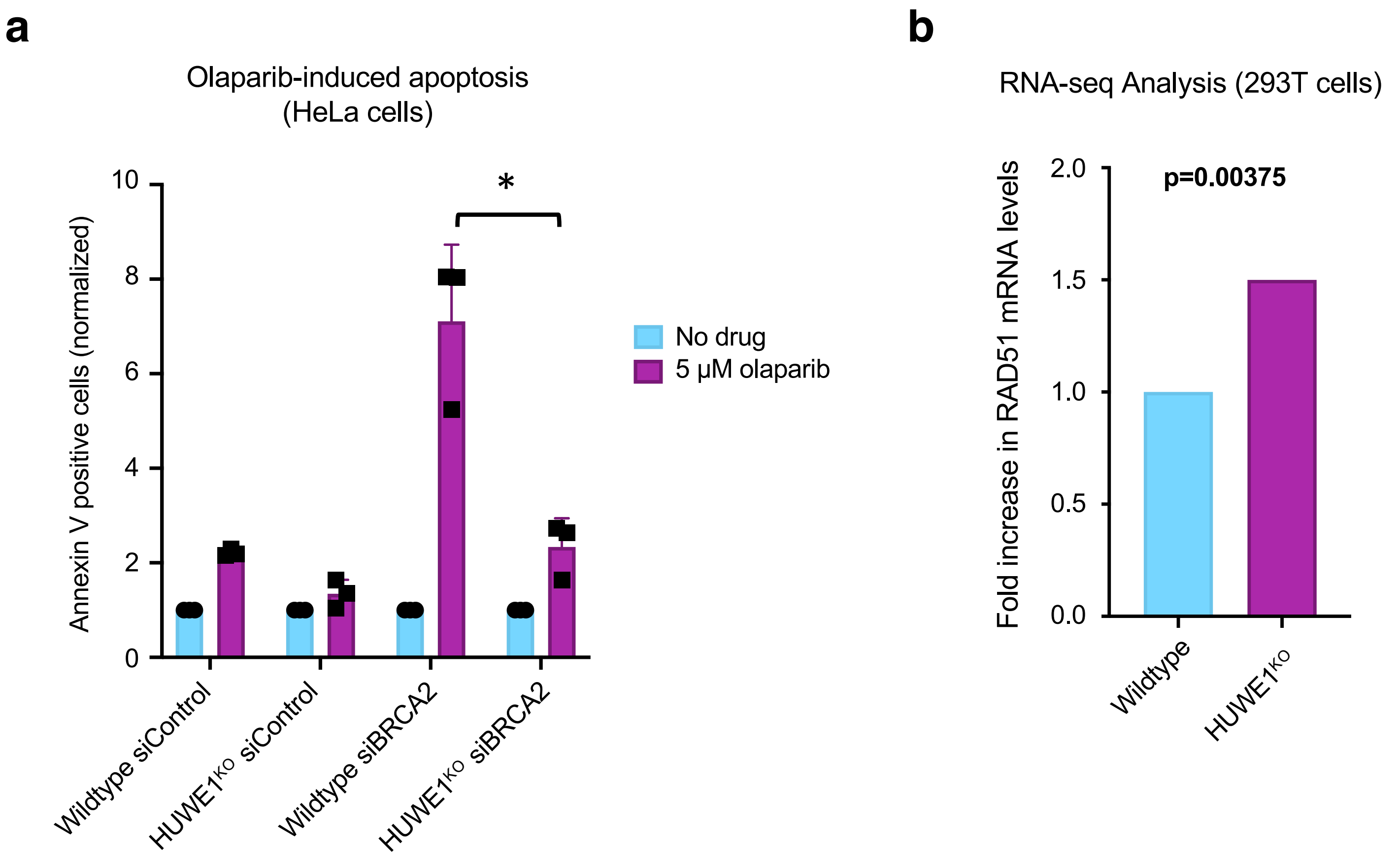

Supplementary Fig. 7. Impact of HUWE1-knockout on olaparib-induced apoptosis and RAD51 levels. a. HUWE1-knockout cells show reduced olaparib-induced apoptosis upon BRCA2 depletion. The average of three experiments performed after 72 hours of treatment is presented, with standard deviations shown as error bars. Asterisks indicate statistical significance (t-test, two-tailed, unequal variance). **b.** RNA-seq analyses show that RAD51 mRNA levels are increased in HUWE1-knockout cells. The mean RAD51 FPKM (Fragments Per Kilobase of transcript per Million mapped reads) values were calculated from 5 biological replicates in HUWE1-knockout cells (47.6707, 34.9944, 53.2581, 30.1317 and 33.0322) and 3 biological replicates in wildtype cells (25.487, 25.6783 and 28.3277). The fold increase shown in the graph was obtained by normalizing the mean value of the HUWE1 knockout cells (39.8174) to that of the wildtype samples (26.4976). The p-value indicated was calculated using the Wald test. Source data are provided as a Source Data file.

Supplementary Figure 8

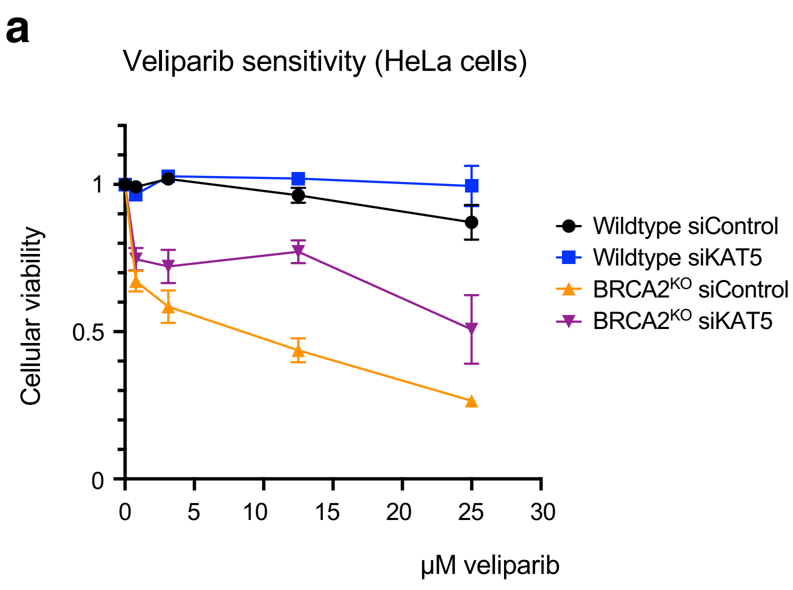

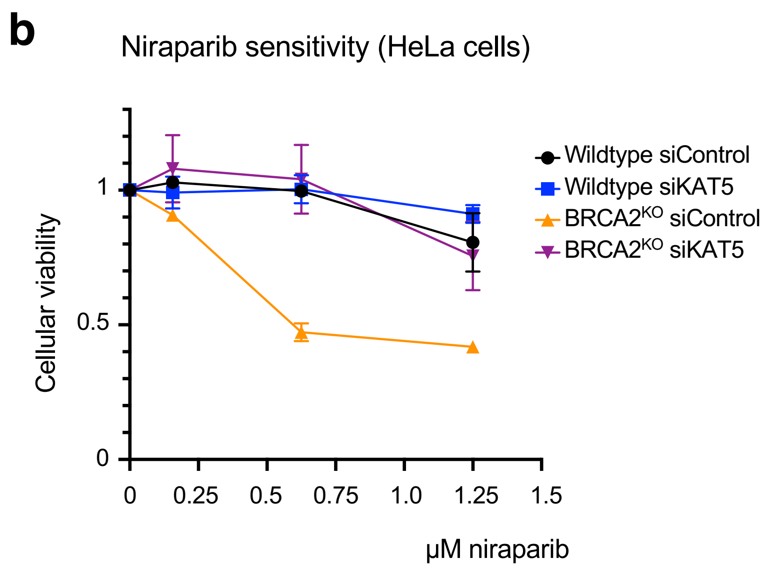

Supplementary Fig. 8. KAT5 depletion rescues the sensitivity of BRCA2-deficient cells to multiple PARP inhibitors. Knockdown of KAT5 rescues the veliparib (**a**) and niraparib (**b**) sensitivity of BRCA2-knockout HeLa cells in cellular viability assays. The graphs present the average of three experiments performed after 72 hours of treatment, with standard deviations shown as error bars. Source data are provided as a Source Data file.

Supplementary Figure 9

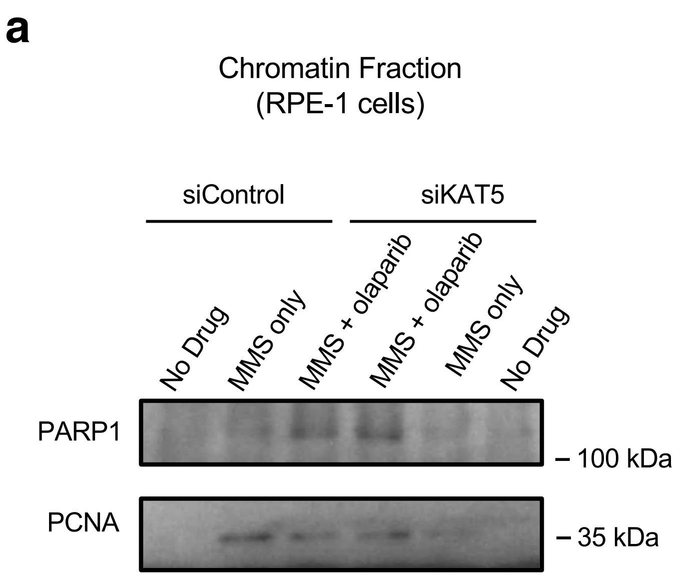

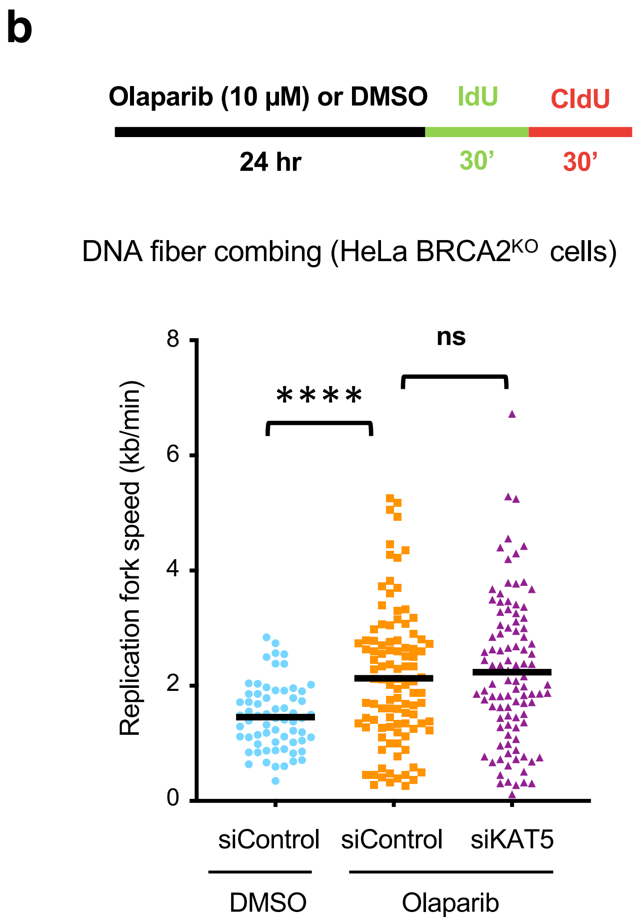

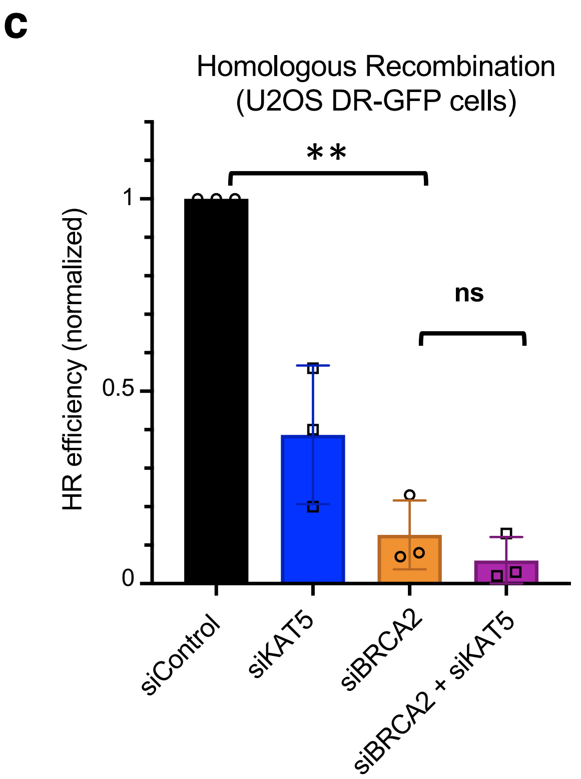

Supplementary Fig. 9. KAT5 depletion does not affect other proposed mechanisms of PARPi cytoxicity. a. A chromatin fractionation experiment shows that while MMS (0.01%) and olaparib (1μM) co-treatment for 3 hours induces trapping of PARP1 on the chromatin, this effect is not rescued by KAT5 depletion. This is an independent replicate of the experiment shown in Fig. 5a. **b.** DNA fiber assay performed in HeLa BRCA2 knockout cells demonstrates that while olaparib treatment increases replication fork speed, this phenotype is not affected by KAT5 depletion. Olaparib (10μM) was added for 24 hours prior to incubation with the thymidine analogs. Horizontal bars reflect the means of replication fork speeds calculated from measurements of CldU tracts. At least 65 fibers were quantified from one experiment. Asterisks indicate statistical significance (t-test, two-tailed, unequal variance). **c.** KAT5 depletion does not rescue the homologous recombination defect caused by BRCA2 depletion as shown using a DR-GFP assay. The averages of three experiments are shown, with standard deviations as error bars. Statistical significance is indicated by asterisks (t-test, two-tailed, unequal variance). Source data are provided as a Source Data file.

Supplementary Figure 10

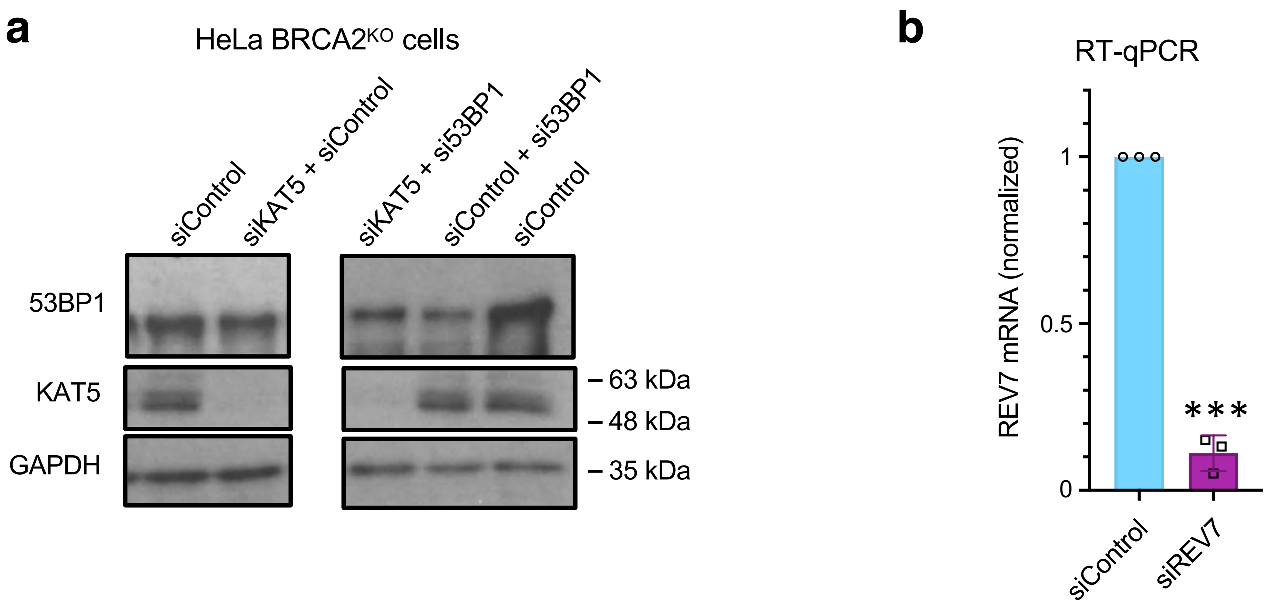

Supplementary Fig. 10. Validation of gene knockdowns. a. Western blot showing that 53BP1 is depleted by the siRNA oligonucleotides employed, and that KAT5 depletion is equivalent when depleted alone or in combination with 53BP1. **b.** Quantitative PCR assay demonstrating the efficacy of the siRNA oligonucleotide targeting REV7. Data shown is the average of three experiments, normalized to the siControl condition, with standard deviations shown as error bars. Statistical significance is indicated by asterisks (t-test, two-tailed, unequal variance). Source data are provided as a Source Data file.

Supplementary Figure 11

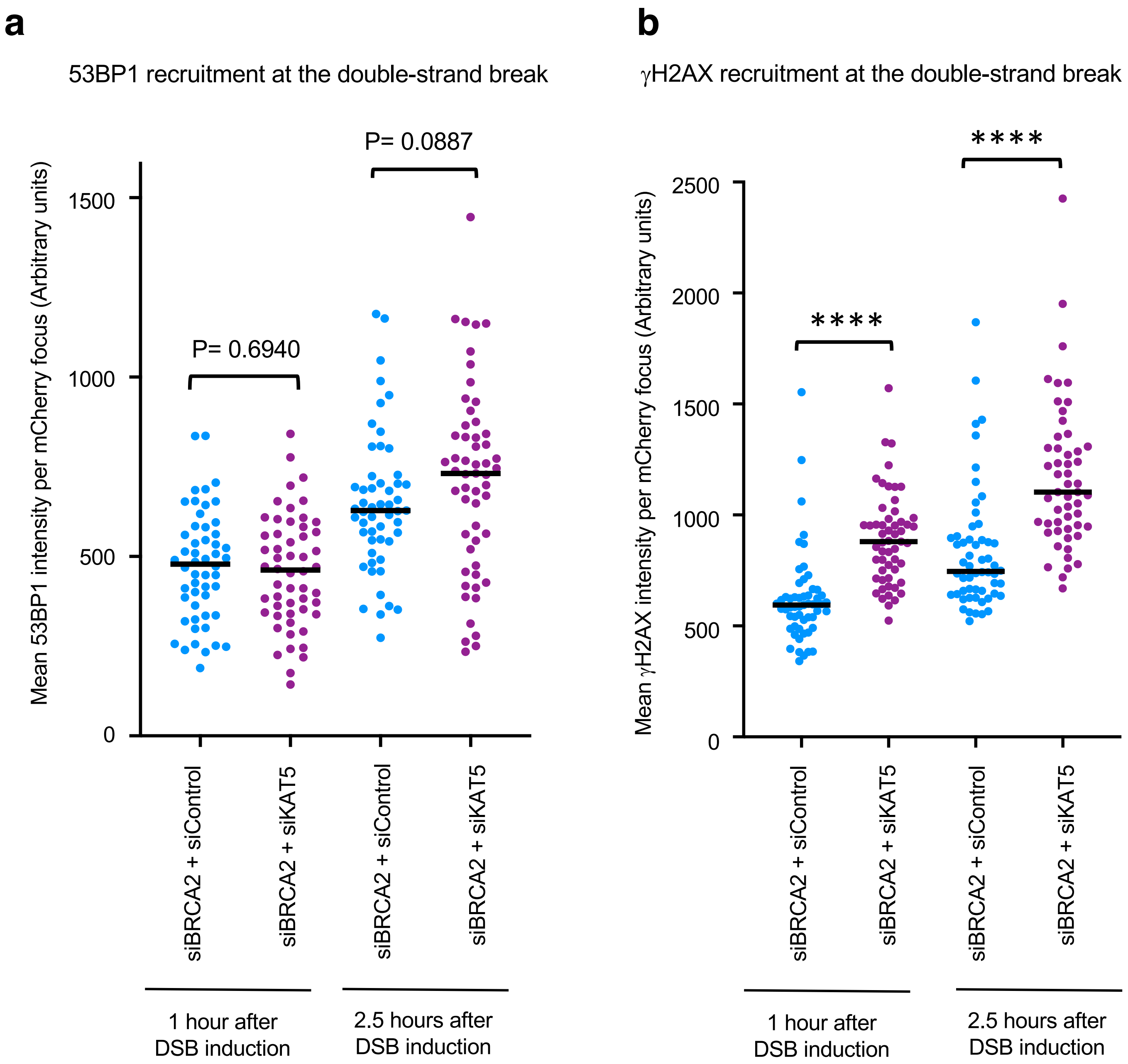

Supplementary Fig 11. Kinetics of repair protein accumulation at sites of DSB induction in BRCA2-depleted cells. Immunofluorescence experiment in U2OS-DSB cells showing mean 53BP1 **(a)** and γH2AX **(b)** intensity at DSB sites (marked by mCherry signal), at the indicated time points after treatment with the agents that initiate DSB induction. At least 50 cells were analyzed for each condition, from one experiment. The median values, and the statistical significance (Mann-Whitney test, two-sided) are shown. Source data are provided as a Source Data file.

Supplementary Figure 12

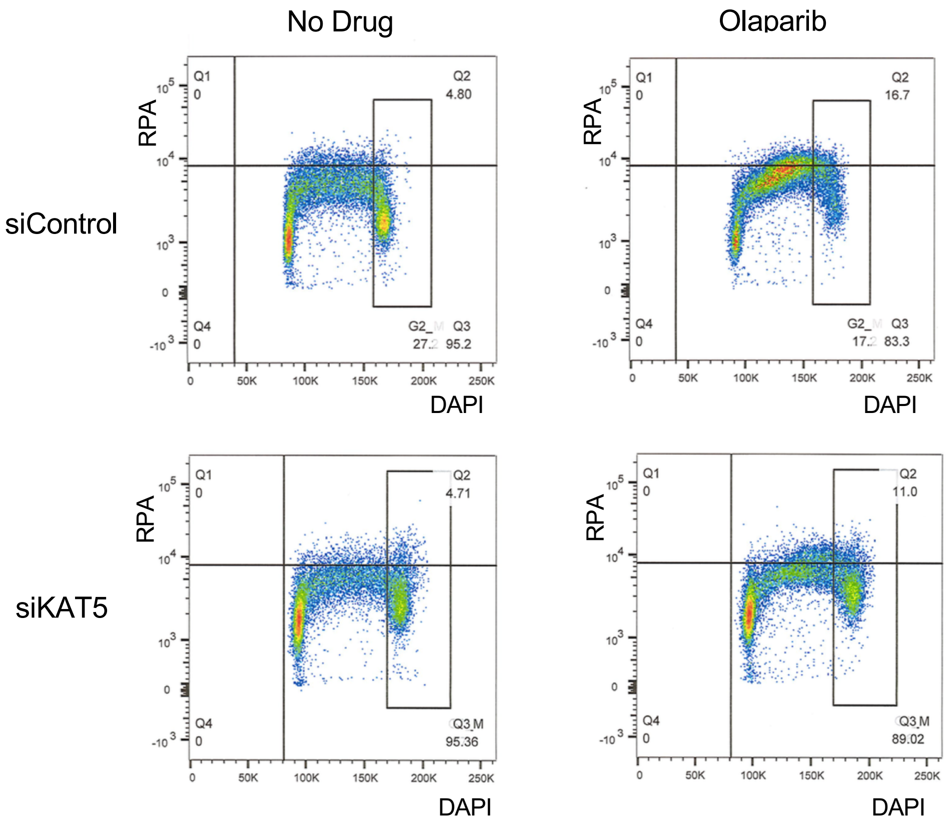

Supplementary Fig. 12. Flow cytometry dot plot of DAPI versus RPA staining intensity. The dot plots illustrate the gating (inset boxes) used to produce Fig. 6b. Cells in Quadrant 2 (Q2) were considered RPA-positive, and cells in Quadrant 3 (Q3) RPA-negative; this cutoff was determined based on the “No treatment” condition and was kept constant across all samples. Approximately 20,000 events per sample from a single experiment are shown, and similar results were obtained across 3 experiments.

Supplementary Figure 13

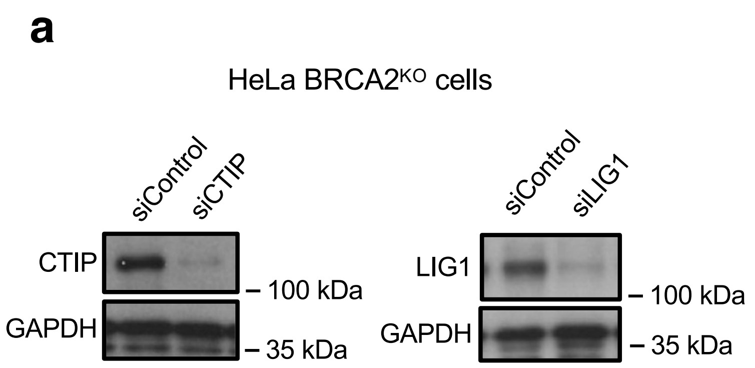

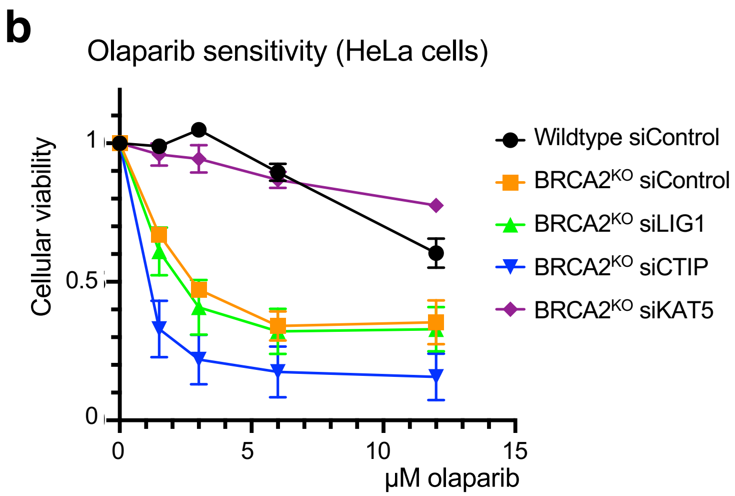

Supplementary Fig. 13. Depletion of CTIP or LIG1 does not rescue the olaparib sensitivity of BRCA2-knockout HeLa cells. a. The efficacy of the siRNA oligonucleotides targeting CTIP and LIG1 are shown using western blot. **b.** Cellular viability assays demonstrate that depletion of CTIP or LIG1 is not sufficient to cause PARPi resistance in BRCA2-knockout HeLa cells. The averages of three experiments are shown, with standard deviations as error bars. Source data are provided as a Source Data file.
