# Supplementary material for: Identification of regulators of poly-ADP-ribose polymerase inhibitor response through complementary CRISPR knockout and activation screens
Source: Nat Commun. 2020 Nov 30;11:6118. doi: 10.1038/s41467-020-19961-w (PMC7704667; doi:10.1038/s41467-020-19961-w)
Supplement: Supplementary file 3 — Description of Additional Supplementary Files [file 41467_2020_19961_MOESM3_ESM.pdf]

Description of Additional Supplementary Files

File Name: Supplementary Data 1
Description: **Gene ranking and sgRNA read counts for the CRISPR knockout screen for olaparib sensitivity in wildtype cells.** All genes targeted by the library are ranked according to the MAGeCK negative selection score. The individual sgRNA sequences, their scores, and the read counts in each of the replicate screens are also presented.

File Name: Supplementary Data 2
Description: **Gene ranking and sgRNA read counts for the CRISPR knockout screen for olaparib resistance in BRCA2^{KO} cells.** All genes targeted by the library are ranked according to the MAGeCK positive selection score. The individual sgRNA sequences, their scores, and the read counts in each of the replicate screens are also presented.

File Name: Supplementary Data 3
Description: **Gene ranking and sgRNA read counts for the CRISPR activation screen for olaparib resistance in BRCA2^{KO} cells.** All genes targeted by the library are ranked according to the MAGeCK positive selection score. The individual sgRNA sequences, their scores, and the read counts in each of the replicate screens are also presented.
