# Supplementary material for: Identification of regulators of poly-ADP-ribose polymerase inhibitor response through complementary CRISPR knockout and activation screens
Source: Nat Commun. 2020 Nov 30;11:6118. doi: 10.1038/s41467-020-19961-w (PMC7704667; doi:10.1038/s41467-020-19961-w)
Supplement: Supplementary file 7 — Reporting Summary [file 41467_2020_19961_MOESM7_ESM.pdf]

nature research

Corresponding author(s): George-Lucian Moldovan

Last updated by author(s): Oct 15, 2020

Reporting Summary

Nature Research wishes to improve the reproducibility of the work that we publish. This form provides structure for consistency and transparency in reporting. For further information on Nature Research policies, see our Editorial Policies and the Editorial Policy Checklist.

Statistics

For all statistical analyses, confirm that the following items are present in the figure legend, table legend, main text, or Methods section.

n/a	Confirmed	
☐	☒	The exact sample size (n) for each experimental group/condition, given as a discrete number and unit of measurement
☐	☒	A statement on whether measurements were taken from distinct samples or whether the same sample was measured repeatedly
☐	☒	The statistical test(s) used AND whether they are one- or two-sided<br>*Only common tests should be described solely by name; describe more complex techniques in the Methods section.*
☒	☐	A description of all covariates tested
☒	☐	A description of any assumptions or corrections, such as tests of normality and adjustment for multiple comparisons
☐	☒	A full description of the statistical parameters including central tendency (e.g. means) or other basic estimates (e.g. regression coefficient) AND variation (e.g. standard deviation) or associated estimates of uncertainty (e.g. confidence intervals)
☐	☒	For null hypothesis testing, the test statistic (e.g. F, t, r) with confidence intervals, effect sizes, degrees of freedom and P value noted<br>*Give P values as exact values whenever suitable.*
☒	☐	For Bayesian analysis, information on the choice of priors and Markov chain Monte Carlo settings
☒	☐	For hierarchical and complex designs, identification of the appropriate level for tests and full reporting of outcomes
☒	☐	Estimates of effect sizes (e.g. Cohen's d, Pearson's r), indicating how they were calculated

Our web collection on statistics for biologists contains articles on many of the points above.

Software and code

Policy information about availability of computer code

Data collection	1) Microscopy (Immunofluorescence, DNA Fiber Combing, Comet assay): LASX 3.3.0.16799<br>2) Flow Cytometry: BD FACSDiva 8.0.1
Data analysis	1) DNA Fiber Combing: LASX 3.3.0.16799<br>2) Immunofluorescence: ImageJ 1.52p<br>3) Flow Cytometry: FlowJo v10<br>4) Comet Assay analyses: CometScore 2.0<br>5) Statistics and Data Figure Panels: GraphPad Prism 8 and Microsoft Excel 15<br>6) CRISPR screen analyses: MAGeCK 0.5.6 (obtained from https://sourceforge.net/p/mageck/wiki/libraries/)<br>7) Survival analyses: R survival package version 2.41-3

For manuscripts utilizing custom algorithms or software that are central to the research but not yet described in published literature, software must be made available to editors and reviewers. We strongly encourage code deposition in a community repository (e.g. GitHub). See the Nature Research guidelines for submitting code & software for further information.

Data

Policy information about availability of data

All manuscripts must include a data availability statement. This statement should provide the following information, where applicable:

- Accession codes, unique identifiers, or web links for publicly available datasets
- A list of figures that have associated raw data
- A description of any restrictions on data availability

Data supporting the findings of this manuscript are available from the corresponding author upon reasonable request. Read counts for all screens are provided in

Supplementary Data files. The source data underlying Figs. 1b, d, f, 2a-c, 3a-f, 4a-d, 5a-d, 6a-c, 7a-c, and Supplementary Figs. 1a-b, 2a-b, 3a-b, 4a-b, 6a-f, 7a-b, 8a-b, 9a-c, 10a-b, 11a-b, 13a-b are provided as a Source Data file.
Genomic and transcriptomic data for the OV-AU cohort was obtained from https://dcc.icgc.org/projects/OV-AU and https://xenabrowser.net/datapages/?hub=https://tcga.xenahubs.net:443.

Field-specific reporting

Please select the one below that is the best fit for your research. If you are not sure, read the appropriate sections before making your selection.

☒ Life sciences ☐ Behavioural & social sciences ☐ Ecological, evolutionary & environmental sciences

For a reference copy of the document with all sections, see nature.com/documents/nr-reporting-summary-flat.pdf

Life sciences study design

All studies must disclose on these points even when the disclosure is negative.

Sample size	No statistical method was used to predetermine sample size. Sample size was determined based on previous experiments and relevant literature in the field (eg Sanson et al., PMID: 30575746; Lemacon et al, PMID: 29038425). Statistical significance was obtained with these sample size.
Data exclusions	No data was excluded from the analysis.
Replication	The three CRISPR screens were performed in duplicate. Western blot experiments, DNA fiber assays, immunofluorescence and comet assays were performed at least twice (with the exception of Supplementary Fig. 9b and 11, which were only done once). All results were reproducible. The experiment in Supplementary Fig. 9b was only performed once because it is a negative result and it addresses an issue not directly linked to the theme of the manuscript and is thus inconsequential. The experiment in Supplementary Fig. 11 was only done once because of time constraints during the revision. The drug sensitivity assays were replicated independently for the number of times indicated in the figure legends. All attempts at replication were successful.
Randomization	Samples were randomly allocated into experimental groups. Samples were processed and analyzed in random order.
Blinding	For imaging-based assays, samples were tested at least once under blinding conditions, and results were reproduced. Blinding is less feasible for the other assays (such as those western blot-based) and was not performed. However, these results were reproduced independently by at least two different authors.

Reporting for specific materials, systems and methods

We require information from authors about some types of materials, experimental systems and methods used in many studies. Here, indicate whether each material, system or method listed is relevant to your study. If you are not sure if a list item applies to your research, read the appropriate section before selecting a response.

Materials & experimental systems

n/a	Involved in the study	
☐	☒	Antibodies
☐	☒	Eukaryotic cell lines
☒	☐	Palaeontology and archaeology
☒	☐	Animals and other organisms
☒	☐	Human research participants
☒	☐	Clinical data
☒	☐	Dual use research of concern

Methods

n/a	Involved in the study	
☒	☐	ChIP-seq
☐	☒	Flow cytometry
☒	☐	MRI-based neuroimaging

Antibodies

Antibodies used	Antibodies used for western blots were: ABCB1 (Santa Cruz Biotechnology, sc-13131), GAPDH (Santa Cruz Biotechnology, sc-47724), KAT5 (Santa Cruz Biotechnology, sc-166323), HUWE1 (Bethyl, A300-486A), Vinculin (Santa Cruz Biotechnology, sc-25336), 53BP1 (Bethyl, A300-272A), CTIP (Santa Cruz Biotechnology, sc-271339), LIG1 (Bethyl, A301-136A), PARP1 (Cell Signaling, 9542), PCNA (Cell Signaling, 2586), Cas9 (BioLegend 844302), Histone H2B (Cell Signaling, 12364), Lamin B1 (Abcam ab16048), RAD51 (Santa Cruz Biotechnology, sc-8349). All antibodies were used at a dilution of 1:500. Uncropped scans of all blots, including molecular weight markers, are provided as a Source Data file.<br>Antibodies used for immunofluorescence were: 53BP1 (Bethyl A300-272A), gH2AX (Bethyl A300-081A), AlexaFluor 488 (Invitrogen A11008).<br>For DNA fiber staining, primary antibodies used were: Anti-BrdU BU1/75 (Abcam, 6326) for detection of CldU; Anti-BrdU B44 (BD, 347580) for detection of IdU; Anti-single stranded DNA (Millipore Sigma, MAB3034) for detection of DNA. Secondary antibodies used were: Anti-mouse Cy3.5 preadsorbed (Abcam, 6946); Anti-rat Cy5 preadsorbed (Abcam, 6565); Anti-rabbit BV480-conjugated (BD

Biosciences, 564879).

Validation

ABCB1: https://datasheets.scbt.com/sc-13131.pdf and Figure 2a
GAPDH: https://www.scbt.com/p/gapdh-antibody-0411
KAT5: https://www.scbt.com/p/tip60-antibody-c-7 and Supplementary figure 6a
HUWE1: https://www.bethyl.com/product/A300-486A and Supplementary figure 6c
Vinculin: https://www.scbt.com/p/vinculin-antibody-h-10
53BP1: https://www.bethyl.com/product/A300-272A
CTIP: https://www.scbt.com/p/ctip-antibody-d-4 and Supplementary figure 13a
LIG1 : https://www.bethyl.com/product/A301-136A and Supplementary figure 13a
PARP1: https://www.cellsignal.com/products/primary-antibodies/parp-antibody/9542
PCNA: https://www.cellsignal.com/products/primary-antibodies/pcna-pc10-mouse-mab/2586
Cas9: https://www.biolegend.com/en-us/products/purified-anti-crispr-cas9-antibody-11774 and Supplementary figure 3a
Histone H2B: https://www.cellsignal.com/products/primary-antibodies/histone-h2b-d2h6-rabbit-mab/12364
Lamin B1: https://www.abcam.com/lamin-b1-antibody-nuclear-envelope-marker-ab16048.html
RAD51: https://www.scbt.com/p/rad51-antibody-h-92 and Figure 4c
gH2AX: https://www.bethyl.com/product/A300-081A
Anti-BrdU BU1/75: https://www.abcam.com/brdu-antibody-bu175-icr1-proliferation-marker-ab6326.html
Anti-BrdU B44: https://www.bdbiosciences.com/us/applications/research/apoptosis/purified-antibodies/purified-mouse-anti-brdu-b44/p/347580
Anti-single stranded DNA: https://www.emdmillipore.com/US/en/product/Anti-DNA-Antibody-single-stranded-clone-16-19,MM_NF-MAB3034

Eukaryotic cell lines

Policy information about cell lines

Cell line source(s)

HeLa, U2OS, and RPE cells were obtained from ATCC. DLD1 cells were obtained from Dr. Robert Brosh (National Institute on Aging, NIH); the original source of this cell line is Horizon Discovery (HD105-007). U2OS-DSB reporter cells were obtained from Dr. Roger Greenberg (University of Pennsylvania) who created this cell line. U2OS DR-GFP cells were obtained from Dr. Jeremy Stark (City of Hope National Medical Center, Duarte, CA) who created this cell line.

Authentication

Authentication was performed regularly based on morphology and gene/protein expression (in case of genetic alterations)

Mycoplasma contamination

If found to be contaminated with mycoplasma, cell lines were treated with plasmocin.

Commonly misidentified lines
(See ICLAC register)

None of the cell lines used are listed on the ICLAC register version 10

Flow Cytometry

Plots

Confirm that:

☒ The axis labels state the marker and fluorochrome used (e.g. CD4-FITC).

☒ The axis scales are clearly visible. Include numbers along axes only for bottom left plot of group (a 'group' is an analysis of identical markers).

☒ All plots are contour plots with outliers or pseudocolor plots.

☒ A numerical value for number of cells or percentage (with statistics) is provided.

Methodology

Sample preparation

We used cell lines, thus no special sample preparation was needed.

Instrument

BD FACSCanto 10

Software

Data collection: BD FACSDiva 8.0.1
Data analyses: FlowJo v10

Cell population abundance

We used cell lines, thus population abundance is not an issue.

Gating strategy

For the quantification of RPA-positive cells by flow cytometry, Cells were first gated using FSC-A/SSC-A, than G2/M cells were gated based on DAPI signal. The parameters were kept the same between samples. Supplementary Fig. 12 presents the gating strategy.

☒ Tick this box to confirm that a figure exemplifying the gating strategy is provided in the Supplementary Information.
